# Adolescent-onset anti-MDA5 antibody-positive juvenile dermatomyositis with rapidly progressive interstitial lung disease and spontaneous pneumomediastinum: a case report and literature review

**DOI:** 10.1186/s12969-021-00595-1

**Published:** 2021-06-30

**Authors:** Tsz-Wing Yeung, Kai-Ning Cheong, Yu-Lung Lau, Kei-Chiu Niko Tse

**Affiliations:** 1 Department of Paediatrics and Adolescent Medicine, Tuen Mun Hospital, Tuen Mun, Hong Kong SAR; 2 Department of Paediatrics and Adolescent Medicine, Hong Kong Children’s Hospital, Kowloon Bay, Hong Kong SAR; 3 Department of Paediatrics and Adolescent Medicine, The University of Hong Kong, Pok Fu Lam, Hong Kong SAR; 4 Department of Paediatrics and Adolescent Medicine, Queen Mary Hospital, Pok Fu Lam, Hong Kong SAR

**Keywords:** Juvenile dermatomyositis, Anti-melanoma differentiation-associated gene 5 antibody, Interstitial lung disease, Pneumomediastinum, Myositis-specific antibodies

## Abstract

**Background:**

Dermatomyositis with positive anti-melanoma differentiation-associated gene 5 (anti-MDA5) antibody has a distinct phenotype associated with small hand joint arthritis, mucocutaneous ulceration, palmar papules and less muscle involvement. It is also associated with increased risk of rapidly progressive interstitial lung disease (RP-ILD) and has a high mortality rate in adults. There is evidence that cases complicated with spontaneous pneumomediastinum (PNM) have an increase in mortality. While most of the evidence for this rare disease is derived from the adult literature, we report a case diagnosed in an adolescent complicated with both RP-ILD and PNM with a good outcome after aggressive immunosuppressive therapy. Our case also illustrates the potential challenges in diagnosis of this condition in the setting of non-specific clinical manifestations, the need for a high index of suspicion, and the importance of testing for myositis-specific antibodies (MSA) early to aid in diagnosis given the risk of rapid progression in these patients.

**Case presentation:**

A 16-year-old Chinese female presented with fever and cough for 1 day, and finger swelling for 3 weeks. Physical examination revealed arthritis of fingers and wrists, ulcers and palmar papules over fingers, hyperpigmentation of interphalangeal joints, and rash over the neck. The diagnosis of dermatomyositis was made 1 month later with the onset of malar rash, Gottron’s papules, calcinosis and myalgia. The diagnosis was supported by the presence of anti-MDA5 antibody and evidence of inflammatory myopathy on magnetic resonance imaging. In retrospect, she already had interstitial lung disease at first presentation manifested as cough and opacity on chest radiograph, which was later confirmed with chest computed tomography. She was treated according to adult guidelines with steroid and calcineurin inhibitor. Her disease was resistant to initial therapy and was complicated by RP-ILD and spontaneous PNM. Intensive immunosuppressive therapy including cyclophosphamide and rituximab were required to induce remission.

**Conclusions:**

Recognition of distinct clinical features of anti-MDA5 antibody-positive dermatomyositis and testing for MSA is crucial in patients with skin ulceration and abnormal pulmonary findings of unknown etiology, as prompt diagnosis with early aggressive treatment and anticipation of complications could make a difference in the outcome of this disease with high mortality.

## Background

Juvenile dermatomyositis (JDM) is a systemic inflammatory disease characterized by typical cutaneous lesions including Gottron’s papules and heliotrope rash, and proximal muscle weakness with onset before age 18. It is a rare disease that affects 2–4 per million of children each year [1]. The anti-melanoma differentiation-associated gene 5 (anti-MDA5) antibody was identified in 2005 to be associated with clinically amyopathic dermatomyositis (CADM) and rapidly progressive interstitial lung disease (RP-ILD) in adults [2, 3]. CADM is a type of dermatomyositis with predominant cutaneous lesions without muscle weakness, although laboratory or radiological evidence of myositis can be present [4]. RP-ILD is defined as progressive interstitial lung disease (ILD) within 3 months of the onset of respiratory symptoms [5]. Practitioners are becoming increasingly aware of this disease entity and the need to promptly diagnose this disorder due to its lower six-month survival rate: 57% in anti-MDA5 antibody-positive dermatomyositis as compared to 98% in those without the antibody [6]. Also, spontaneous pneumomediastinum (PNM) is an important complication to be aware of as there is evidence that it leads to a higher mortality [7]. We report a case of anti-MDA5 antibody-positive JDM with RP-ILD and spontaneous PNM.

## Case presentation

**Fig. 1:**
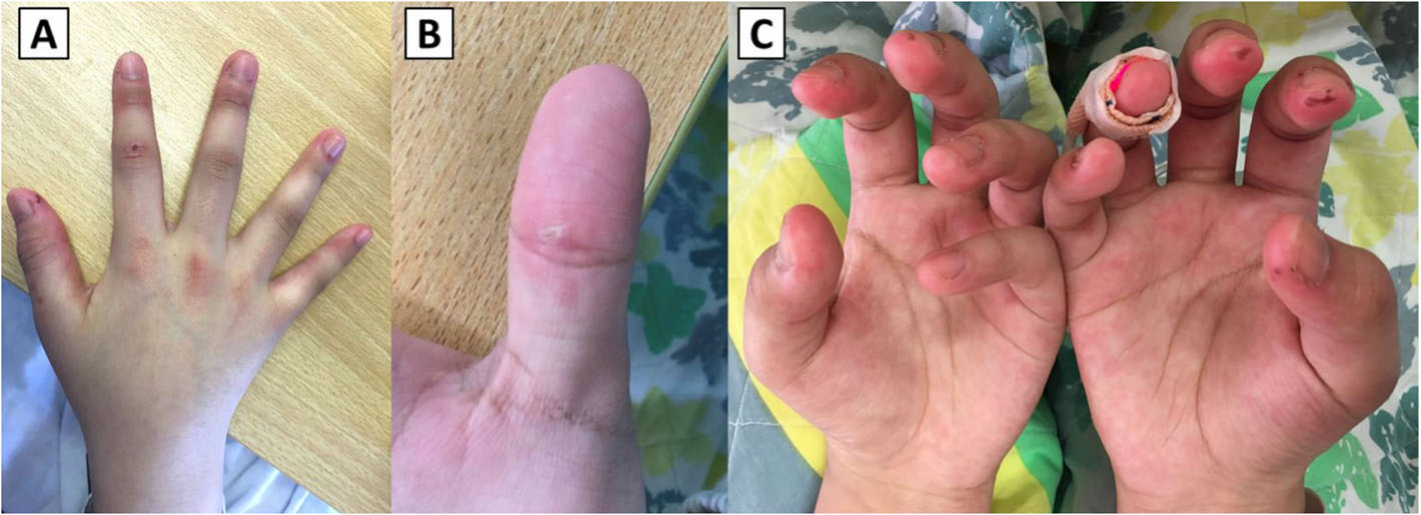
Cutaneous and skeletal features of the patient. **A** Gottron’s papules, interphalangeal joint hyperpigmentation, dactylitis, and skin ulcers over interphalangeal joints and periungual regions. **B** A palmar papule over the thumb. **C** Skin ulcers over finger pulps

A 16-year-old Chinese female with no significant past medical history presented with one-day history of low-grade fever and cough. She also complained of painful swelling of fingers for 3 weeks. Physical examination revealed dactylitis of all fingers, arthritis of both wrists, papules over palmar surface of fingers and small ulcers over interphalangeal joints, periungual regions and finger pulps (Fig. 1). There was hyperpigmentation over the dorsal surface of interphalangeal joints, but no definite Gottron’s papules. Nailfold microhemorrhage was seen using a handheld dermatoscope. She also had erythematous plaques over the neck. She had no muscle weakness. The laboratory tests revealed mildly elevated alanine transaminase (ALT) at 64 U/L (normal range 8–24 U/L) and lactate dehydrogenase (LDH) at 360 U/L (normal range 130–250 U/L). Creatine kinase (CK) level was normal at 129 U/L (normal range 37–173 U/L). C-reactive protein and erythrocyte sedimentation rate were normal. Anti-nuclear antibody was positive with a titer of 1:160 with speckled pattern. Rheumatoid factor was positive at 37 units (positive: > 9 units). Anti-extractable nuclear antibody (anti-ENA) panel revealed positive anti-Ro52 antibody. Other autoantibodies studied including anti-Scl-70, anti-Jo-1, anti-double stranded DNA and anti-cyclic citrullinated peptide were negative. Her chest radiograph showed left perihilar opacity. Although her younger brother was admitted on the same day with mycoplasma pneumonia, her sputum was negative for bacteria including mycoplasma. She was treated for pneumonia and arthritis before being discharged with a course of amoxicillin/clavulanate and naproxen.

**Fig. 2:**
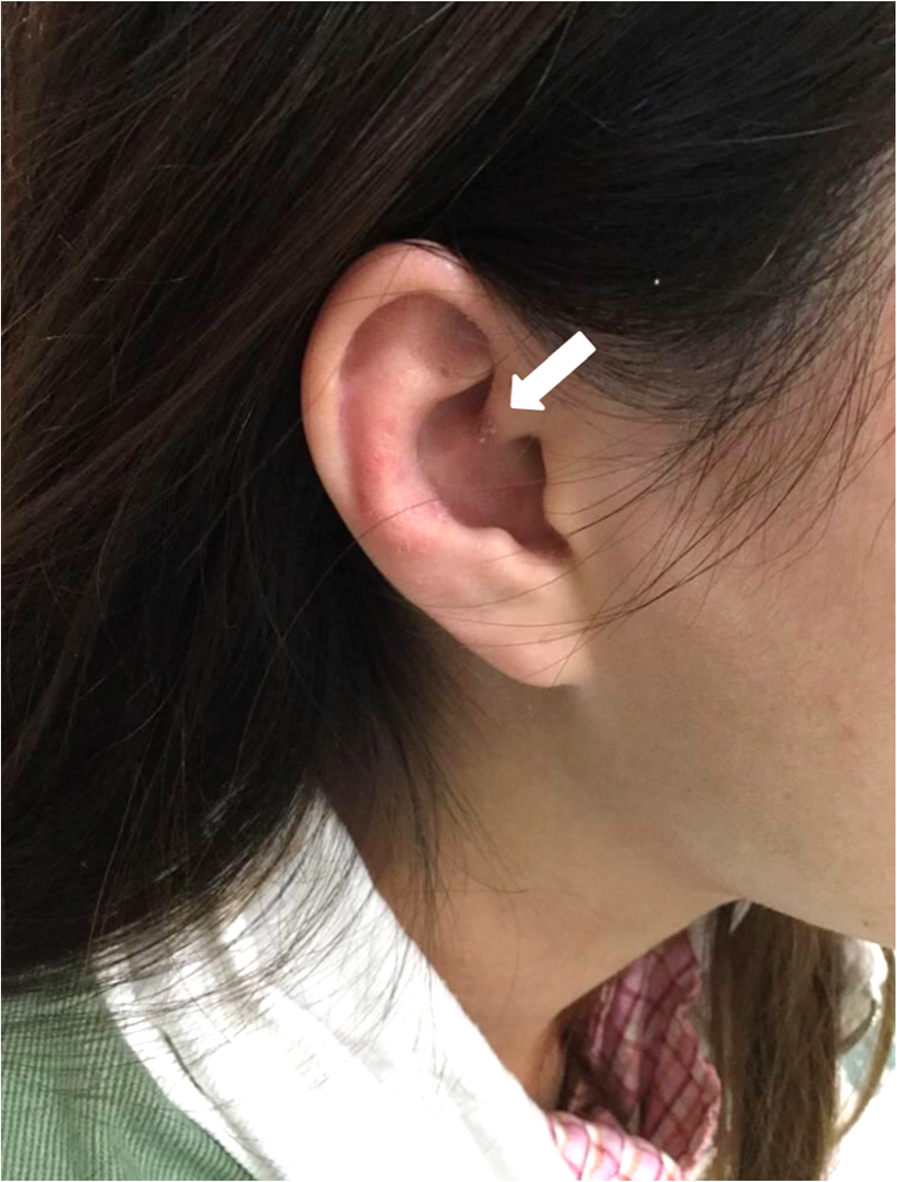
Calcinosis over the external ear

**Fig. 3:**
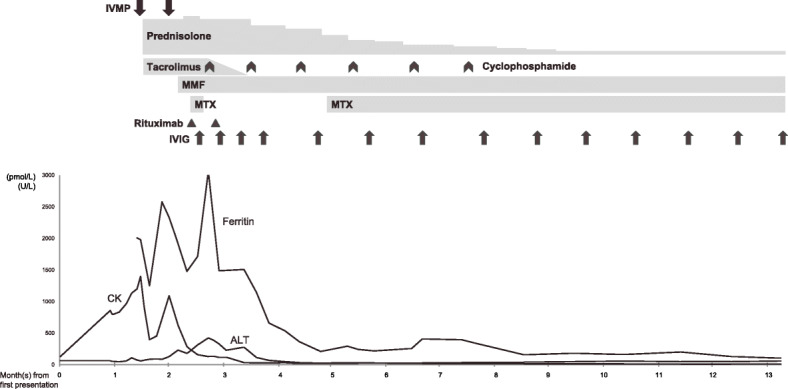
Laboratory results during treatment. IVMP, intravenous methylprednisolone; MMF, mycophenolate mofetil; MTX, methotrexate; IVIG, intravenous immunoglobulin; CK, creatine kinase; ALT, alanine transferase

**Fig. 4:**
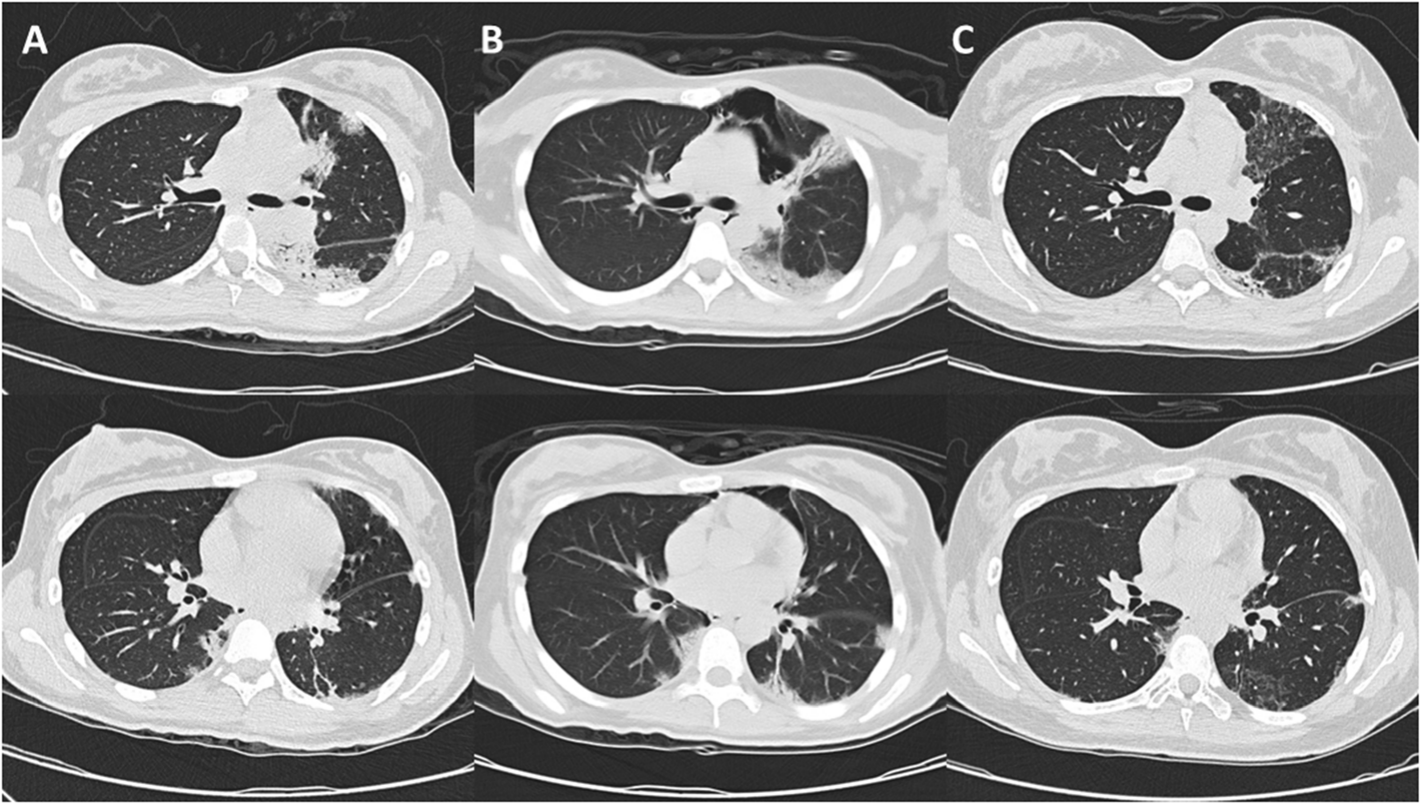
Serial chest computer tomographies of the patient. **A** Subpleural consolidation of lungs at diagnosis. **B** Pneumomediastinum and increase in consolidation 3 weeks after treatment. **C** Resolution of consolidation with fibrosis after rituximab and 3 cycles of cyclophosphamide

Upon follow-up examination 1 month later, she complained of persistent cough and presented new symptoms including muscle pain over proximal limbs and oral ulcers. Examination showed new findings of malar rash, Gottron’s papules (Fig. 1A), calcinosis over external ears (Fig. 2) and bilateral crepitation at lung bases. She had mild proximal weakness over lower limbs with muscle power of Medical Research Council (MRC) grade 4. The Childhood Myositis Assessment Scale (CMAS) score was 42/52 and the Manual Muscle Testing-8 (MMT8) score was 148/150. Blood test showed elevated CK at 858 U/L, LDH at 407 U/L and ferritin at 2006 pmol/L (Fig. 3). Anti-MDA5 antibody was detected in the myositis-specific antibody (MSA) panel, but quantitative test for the antibody was not available. The left perihilar opacity on chest radiograph persisted. Chest computed tomography (CT) showed multiple subpleural and peripherally located consolidations in both lungs (Fig. 4A). No infective organism was isolated from bronchoalveolar lavage. Echocardiogram was normal with no features of pulmonary hypertension. Pulmonary function test showed reduced diffusing capacity for carbon monoxide (DLCO) to 59% of the predicted value. Magnetic resonance imaging (MRI) of pelvis and thighs showed T2-weighted hyperintensity over bilateral pelvic and thigh muscles, consistent with systemic inflammatory myopathy. A diagnosis of JDM with ILD was made without muscle biopsy.

She received a 3-day course of pulse methylprednisolone at 1 g/day, followed by a combination of prednisolone of 1 mg/kg/day and tacrolimus. There was an initial improvement with resolved arthritis and muscle weakness, and decreasing blood CK level (Fig. 3). Two weeks later, the patient developed hoarseness of voice, shortness of breath on exertion, and new vasculitic ulcers over her fingers. Her muscle power deteriorated to MRC grade 3 to 4 over proximal limbs with rebound of blood CK level. Videofluoroscopic swallowing study showed mild pharyngeal dysphagia. A 3-day course of pulse methylprednisolone at 500 mg/day and mycophenolate mofetil (MMF) were added in view of the deterioration.

At week three of treatment, she complained of neck and chest pain. Diffuse neck swelling was noted with crepitus at the anterior neck. She was not in respiratory distress and her oxygen saturation was normal. She was given empirical low flow oxygen supplement. Chest radiograph showed subcutaneous emphysema over the cervical region and PNM. Chest CT showed an increase in consolidative changes in addition to PNM (Fig. 4B). The treatment was intensified with addition of rituximab (1 g each on day 1 and day 15), intravenous immunoglobulin (IVIG) (2 g/kg every 2 weeks for 4 doses, then every 4 weeks for 1 year) and intravenous cyclophosphamide (every 4 weeks for 6 months, cumulative dose 5.75 g/m^2^). Two doses of oral methotrexate (MTX) were given but discontinued due to new elevation of ALT up to 400 U/L. Prescription of tacrolimus was stopped to avoid over-immunosuppression.

**Fig. 5:**
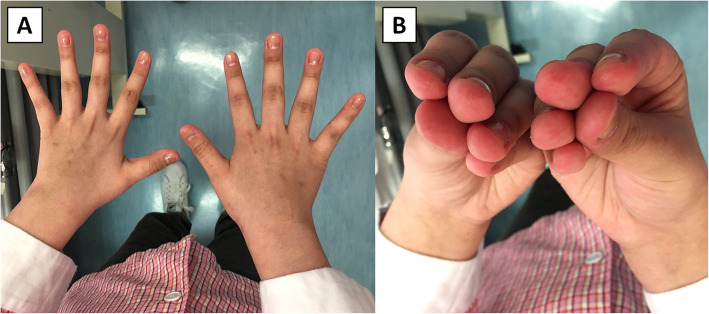
Resolution of cutaneous features after intensification of treatment. **A** Resolution of Gottron’s papules and skin ulcers over interphalangeal joints and periungual regions, with residual interphalangeal joint hyperpigmentation. **B** Resolution of skin ulcers over finger pulps

With aggressive treatment, PNM subsided 1 month later and digital ulcers, cough, hoarseness and muscle weakness were completely resolved after 2 months (Fig. 5). Follow up chest CT after 2 months showed interval resolution of consolidative changes with replacement by fibrosis (Fig. 4C). MTX was reintroduced after ALT normalized, and the dose of prednisolone was tapered. Follow-up pulmonary function test 8 months from diagnosis showed improved DLCO to 80% of the predicted value. At her follow-up 1 year from diagnosis, she had no recurrence while on prednisolone 5 mg daily, maintenance MMF, MTX and monthly IVIG. Her CMAS and MMT8 score were normalized. She did not experience severe side effects such as marrow suppression or severe infection other than elevated intraocular pressure, which was controlled by timolol eye drops.

## Discussion

Dermatomyositis with positive anti-MDA5 antibody has a distinct phenotype in terms of skin, joint, muscle, and lung involvement in both children and adults. It is associated with mucocutaneous ulceration and symmetrical polyarthritis of small joints of the hands. It is also found to be associated with painful palmar papules in adults [8–10]. For muscle involvement, most adult patients are clinically amyopathic, although the distribution varies with the ethnic group where CADM occurred in 82% of Japanese and 45% of Caucasians [6, 11]. CADM was not commonly reported in JDM with anti-MDA5 antibody, but it has a milder muscle involvement compared to those without [8, 12]. For lung involvement, ILD overall occurs rarely in only 8% of JDM [13], while it occurs at a higher frequency in those with anti-MDA5 antibody. In a Japanese study, all 11 out of 35 JDM patients (31%) who possessed the antibody had ILD, of whom 6 had RP-ILD. It was concluded that JDM patients with the antibody were significantly more likely to have RP-ILD [14]. Ethnic differences in the severity of ILD in anti-MDA5 antibody-positive dermatomyositis have been observed, as studies have demonstrated decreased rates of RP-ILD in Caucasian children and adults [8, 11].

It was a diagnostic challenge where our patient presented with non-specific symptoms mimicking other rheumatological conditions. For instance, juvenile idiopathic arthritis was being considered because of the symmetrical small hand joint arthritis with positive rheumatoid factor, however it was less likely because of the presence of other cutaneous features such as skin ulceration. Abnormal nailfold capillaries directed the differential diagnoses to JDM and systemic sclerosis, however the absence of pathognomonic cutaneous features or muscle involvement with a normal CK level gave a false reassurance that JDM had a lower priority. She shared some features of systemic sclerosis including finger ulceration and arthritis. Nevertheless, she did not have skin thickening or other classical features of systemic sclerosis such as Raynaud’s phenomenon and telangiectasia. Calcinosis was not identified at first until the diagnosis of JDM was made, where the calcinosis was small and only present over the external ears. Also, the contact history of pneumonia with an acute onset of fever and cough gave an impression of concomitant pneumonia, instead of pulmonary complications of the underlying rheumatological disease. There was a one-month delay in diagnosis until the onset of pathognomonic cutaneous features and muscle weakness. In retrospect, the patient initially presented with the typical phenotype of anti-MDA5 antibody-positive dermatomyositis with cutaneous ulceration, palmar papules, and small hand joint arthritis. Other than these features, the interphalangeal joint hyperpigmentation should alert one to dermatomyositis. An awareness of less common signs of dermatomyositis and a high index of suspicion are required to make the diagnosis.

The diagnosis of dermatomyositis in our patient was supported by positive anti-MDA5 antibody and evidence of inflammatory myopathy on MRI without performing muscle biopsy and electromyography described in the widely used Bohan and Peter’s criteria [15]. Anti-MDA5 antibody is one of the MSA that is dermatomyositis-specific, which is not found in other inflammatory myopathies or connective tissue disorders [2, 9]. MSA helps distinguish JDM from other connective tissue disorders, which is useful in diagnosis for patients with cutaneous features non-specific to dermatomyositis, while anti-MDA5 antibody is particularly important in patients presenting with skin ulcers of unknown etiology even without muscle weakness as the disease can be subtle on initial presentation. As the field begins to recognize the value of obtaining MSA in patients with JDM, given increasing evidence of distinct phenotypes within the disease, there is an increased call to include MSA results in guidelines. New classification criteria by the European League Against Rheumatism (EULAR)/American College of Rheumatology (ACR) for adult and juvenile idiopathic inflammatory myopathies were recently published that included one MSA – anti-Jo1 antibody [16]. It is expected that other MSA may be incorporated in the classification criteria in the future as it becomes more widely used, and as the field develops more understanding of the distinct disease phenotypes.

From the prognostic standpoint, knowing that a patient is anti-MDA5 antibody positive is valuable because adult studies have demonstrated these patients are at risk for development of RP-ILD which is the major cause of the high mortality. Early screening for pulmonary manifestations allows a window for aggressive treatment to avoid progression to RP-ILD. Other than anti-MDA5 antibody, the coexistence of anti-Ro52 antibody in our patient also predicts the prognosis. A recent study by Xu et al. reported that anti-Ro52 antibody is highly prevalent in anti-MDA5 antibody-positive CADM with ILD, and dual positivity for anti-Ro52 and anti-MDA5 antibody is associated with increased frequency of RP-ILD and lower survival rate in adults [17]. In the pediatric population, Sabbagh et al. suggested that anti-Ro52 antibody is associated with anti-MDA5 antibody, and the presence of anti-Ro52 antibody within the anti-MDA5 subgroup was more strongly associated with ILD [18]. Testing for anti-ENA should be done in suspected cases of dermatomyositis and the combination of positive anti-Ro52 and anti-MDA5 antibody should alert clinicians for close monitoring of pulmonary complications.

Another challenge of JDM with ILD is evidence regarding treatment, due to the rarity of the disease. There is a lack of randomized controlled trials and the current treatment for JDM is largely based on consensus guidelines. The treatment for JDM with ILD or anti-MDA5 antibody is not well established. It could also be attributed to the lack of ILD grading parameters to guide further studies. In 2010, the Childhood Arthritis & Rheumatology Research Alliance (CARRA) in North America reached consensus on the treatment of moderately severe JDM, using a combination of steroid and MTX with or without IVIG [19]; however patients with pulmonary involvement and skin ulceration were excluded. In 2017, the Single Hub and Access point for pediatric Rheumatology in Europe (SHARE) initiative proposed a treatment recommendation for JDM with major organ involvement, using cyclophosphamide in addition to steroid and MTX, and consider the use of IVIG, cyclosporine A, infliximab, rituximab etc. in cases with poor response [20]. However, the decision to intensify treatment is based solely on clinician’s opinion.

Owing to high mortality, treatment for anti-MDA5 antibody-positive JDM should be individualized as early intensification of therapy in those with a poor response may be vital. In adults, the recommended initial treatment for ILD with anti-MDA5 antibody is at least dual therapy with steroid and calcineurin inhibitor with or without cyclophosphamide [21, 22]. We followed this recommendation as the patient was in her adolescence and the fact that there was a lack of pediatric recommendation. It has been debated whether to use cyclophosphamide in view of the risk of gonadotoxicity for young females. However, her disease was resistant to steroid and calcineurin inhibitor with rapid progression of ILD and was complicated by spontaneous PNM. Her lack of initial treatment response and the rapid development of PNM was concerning, especially because literature has reported significantly higher mortality in adult anti-MDA5 antibody-positive dermatomyositis patients with PNM [7]. Her treatment was escalated at that time, and multiple immunosuppressants including cyclophosphamide and rituximab were required to induce disease remission. Rituximab is a chimeric monoclonal anti-CD20 antibody that depletes B-cell. After the initial infusion of rituximab, her CD19+ count dropped from 221 cells/uL to near zero 1 month later, and remain suppressed for 6 months. It is difficult to conclude the efficacy of rituximab as multiple agents were added in proximity. The use of rituximab in RP-ILD with positive anti-MDA5 antibody appeared promising in an adult case series [23], however there are few reports on its use in pediatric patients with the antibody. IVIG was added for immunomodulation and was given for prolonged period to reduce the risk of infection from rituximab-associated hypogammaglobinemia, where her CD19+ count had a slow recovery and had not yet returned to normal at 50 cells/uL 10 months after the infusion. Following disease remission, she received a combination of MMF and MTX as maintenance therapy, considering the poor prognosis suggested by the coexistence of anti-Ro52 and anti-MDA5 antibody, and severe complications with RP-ILD and PNM. She tolerated the treatment without severe infection or major organ dysfunction except for elevated intraocular pressure related to steroid use.

While our patient responded to aggressive immunosuppressive treatment, there is a need for novel mechanism-based treatment in the era of molecular medicine to improve the survival of refractory dermatomyositis. It has been reported that the type I interferon (IFN) pathway is involved in the pathogenesis of juvenile and adult dermatomyositis [24]. Ladislau et al. demonstrated in 2018 that type I IFN pathway activation in vitro reproduces the main dermatomyositis pathological findings including muscle atrophy and vasculopathy, and the pathogenic effects in vitro were abolished by a Janus kinase (JAK) inhibitor ruxolitinib that targets the IFN pathway [25]. Sabbagh et al. in 2019 reported the use of tofacitinib, a JAK inhibitor, leading to clinical improvement within 6 months in two anti-MDA5 antibody-positive JDM refractory to multiple agents including rituximab [26]. Further studies are required to shed light on the use of JAK inhibitor as adjuvant or rescue treatment for this subset of JDM.

## Conclusion

In conclusion, physicians must be aware of the unusual phenotype of anti-MDA5 antibody-positive dermatomyositis. Test for MSA including anti-MDA5 antibody is crucial in patients with skin ulceration and abnormal pulmonary findings of unknown etiology, as early diagnosis and treatment of this rare but severe disease may improve outcomes. Aggressive treatment is warranted especially in patients with PNM or coexistence of anti-Ro52 antibody as they correlate with increased mortality. In view of the poor prognosis of the disease, further studies are necessary to explore the optimal treatment for anti-MDA5 antibody-positive dermatomyositis in the pediatric group.

## Data Availability

Not applicable.

## Abbreviations

Anti-MDA5: Anti-melanoma differentiation-associated gene 5
RP-ILD: Rapidly progressive interstitial lung disease
PNM: Pneumomediastinum
MSA: Myositis-specific antibodies
JDM: Juvenile dermatomyositis
CADM: Clinically amyopathic dermatomyositis
ILD: Interstitial lung disease
ALT: Alanine transaminase
LDH: Lactate dehydrogenase
CK: Creatine kinase
Anti-ENA: Anti-extractable nuclear antibody
MRC: Medical Research Council
CMAS: Childhood Myositis Assessment Scale
MMT8: Manual Muscle Testing-8
CT: Computed tomography
DLCO: Diffusing capacity for carbon monoxide
MRI: Magnetic resonance imaging
MMF: Mycophenolate mofetil
IVIG: Intravenous immunoglobulin
MTX: Methotrexate
IFN: Interferon
JAK: Janus kinase
